# Supplementary material for: Prospective Analysis of the Temporal Relationship between Psychological Distress and Atopic Dermatitis in Female Adults: A Preliminary Study
Source: Healthcare (Basel). 2022 Sep 29;10(10):1913. doi: 10.3390/healthcare10101913 (PMC9601420; doi:10.3390/healthcare10101913)
Supplement: Supplementary file 1 [file healthcare-10-01913-s001.zip › healthcare-1883272-supplementary.pdf]

Title: Prospective analysis of the temporal relationship between psychological distress and atopic dermatitis in female adults

Gurkiran Birdi PhD, Michael Larkin PhD, Rebecca C Knibb PhD

School of Psychology, College of Health and Life Sciences, Aston University, Birmingham, B4 7ET, U.K.

Supplementary Materials

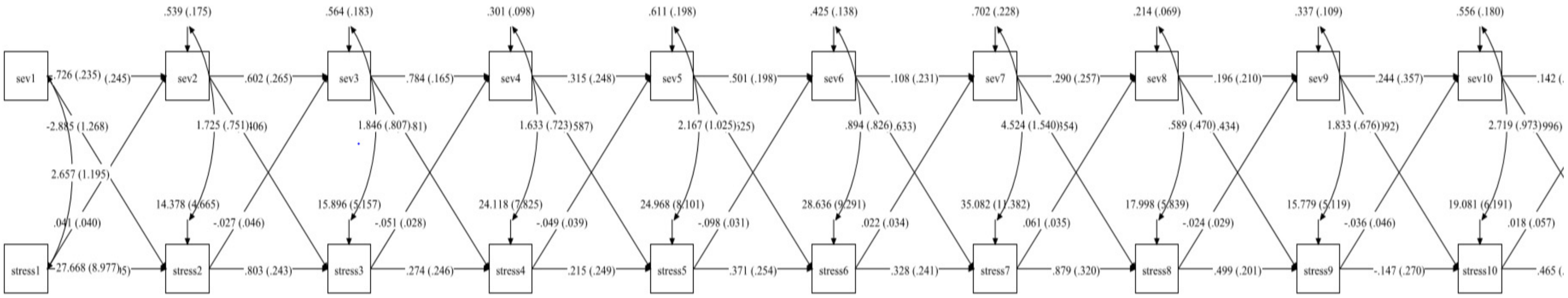

Figure S1: Cross lagged analysis snippet for psychological stress and disease severity for 10 days with β coefficients
